# Exploring host and geographical shifts in transmission of haemosporidians in a Palaearctic passerine wintering in India

**DOI:** 10.1007/s10336-017-1444-9

**Published:** 2017-03-09

**Authors:** Farah Ishtiaq

**Affiliations:** Centre for Ecological Sciences, Indian Institute of Science, Bangalore, 560012 India

**Keywords:** *Acrocephalus dumetorum*, *Haemoproteus*, India, *Plasmodium*, Palaearctic, PCR

## Abstract

This is the first molecular study of avian haemosporidia diversity in wintering populations of the Blyth’s Reed Warbler (*Acrocephalus dumetorum*) in India that explores the extent of host and geographical shifts in transmission areas. In 156 birds, six *Haemoproteus* lineages (37.8%; 95% CI 30.41–45.82%) and one *Plasmodium* lineage (1.9%; 95% CI 0.053–5.6%) were recovered. Of these, two *Haemoproteus* lineages (ACDUM1 and ACDUM5) were detected in resident Himalayan birds, albeit in low frequency with absence of gametocytes in the blood suggesting no transmission from breeding to wintering quarters. In addition, there was no host sharing of local parasite lineages with the wintering populations.

**Electronic supplementary material:**

The online version of this article (doi:10.1007/s10336-017-1444-9) contains supplementary material, which is available to authorized users.

## Zusammenfassung

**Exploring host and geographical shifts in transmission of haemosporidians in a Palaearctic passerine wintering in India** This is the first molecular study of avian haemosporidia diversity in wintering populations of the Blyth’s Reed Warbler (*Acrocephalus dumetorum*) in India that explores the extent of host and geographical shifts in transmission areas. In 156 birds, six *Haemoproteus* lineages (37.8%; 95% CI 30.41–45.82%) and one *Plasmodium* lineage (1.9%; 95% CI 0.053–5.6%) were recovered. Of these, two *Haemoproteus* lineages (ACDUM1 and ACDUM5) were detected in resident Himalayan birds, albeit in low frequency with absence of gametocytes in the blood suggesting no transmission from breeding to wintering quarters. In addition, there was no host sharing of local parasite lineages with the wintering populations.

## Introduction

Blood parasite transmission in the wintering grounds and cross-species infections between migratory and resident birds is considered a cost of migration (Waldenström et al. 2002).

Understanding how parasites disperse between hosts populations is important to predict risk of emerging infectious diseases. The role of migratory birds as bridges in transmission areas has been widely documented, although the research is largely biased towards the Palaearctic-African bird migratory system (e.g., Hellgren et al. 2007). In the Central Asian Flyway, India is a staging and wintering ground for many European passerines; however, we lack information on the transmission or exchange in parasites through migrant avian hosts.

Avian malaria (*Plasmodium* spp.) and other haemosporidians (*Haemoproteus* and *Leucocytozoon* spp., Phylum Apicomplexa, order Haemosporida) are a diverse group of vector-borne blood parasites that have cosmopolitan distributions (Atkinson and van Riper 1991; Valkiūnas 2005). *Plasmodium* is mainly transmitted by culicine mosquitoes while biting midges (Ceratopogonids) and black flies (simulids) are responsible for transmission of *Haemoproteus* and *Leucocytozoon*, respectively.

**Fig. 1:**
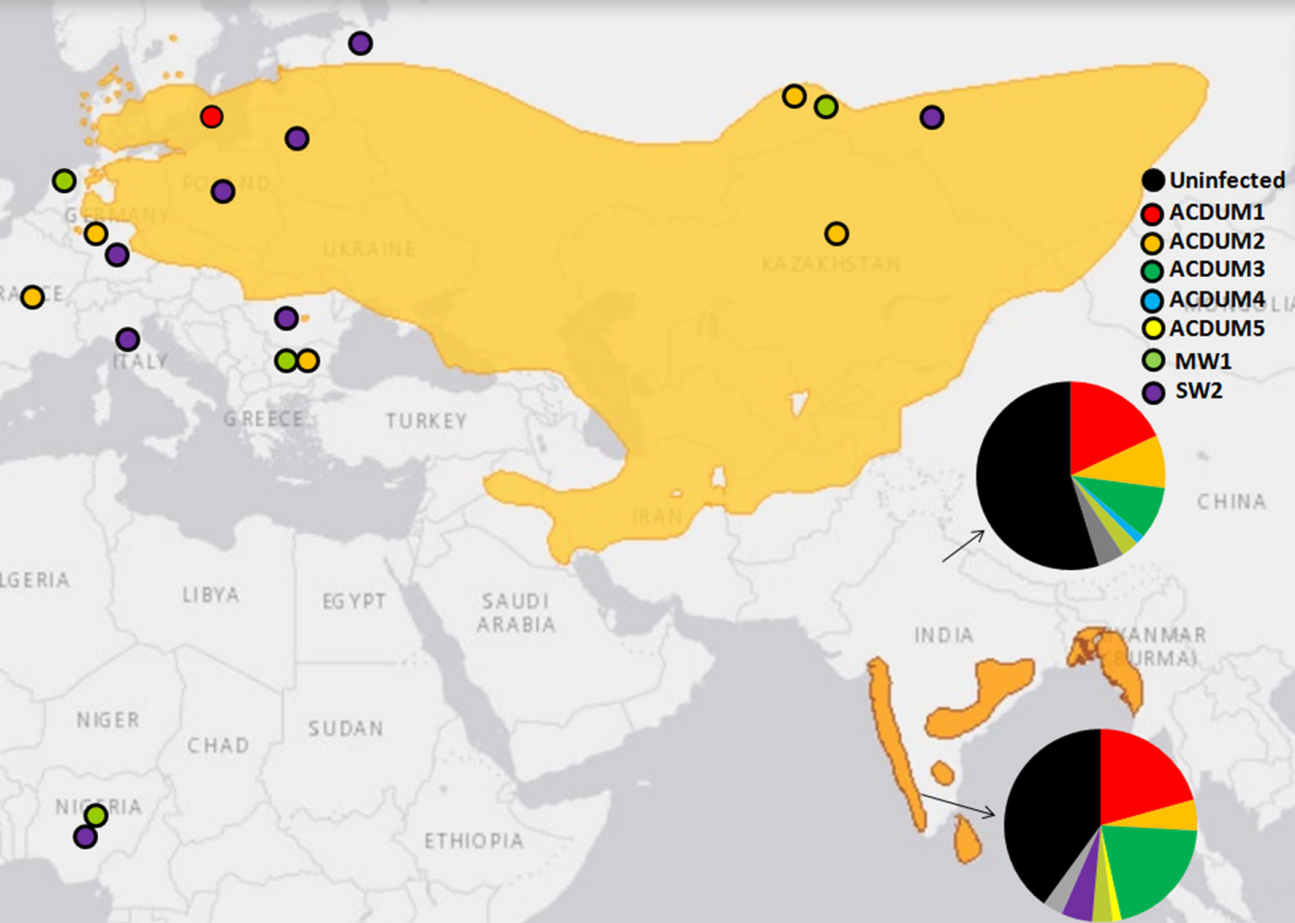
Blyth’s Reed Warbler distribution map with breeding (*light orange*) and wintering (*dark orange*) range. *Pie graphs* indicate prevalence of *Haemoproteus* spp. and *Plasmodium* spp. lineages at sampled states in India. *Colour dots* represent lineages found in Europe and Africa. (map is modified from BirdLife International)

The Blyth’s Reed Warbler (*Acrocephalus dumetorum*) (hereafter BRW) is a Palaearctic passerine that breeds in Eastern Europe, East to Central Russia, South to North Afghanistan and winters exclusively within the Indian subcontinent south of the Himalayas, except for Pakistan and northwest India where it is only a passage migrant with short duration of occurrence during migration (Fig. 1; Ali and Ripley 1973). The BRW occurs in sympatry in breeding and wintering range with many Palaearctic passerines that winter in South Asia. However, the Paddyfield Warbler (*Acrocephalus agricola*) and Common Rosefinch (*Carpodacus erythrinus*) have been the only species studied extensively for avian haemosporidians in the breeding range (Zehtindjiev et al. 2009; Synek et al. 2013).

Here, I present results from the first molecular-based study that investigates the prevalence and diversity of haemosporidians in a Palaearctic passerine wintering over a wide geographical area in India. Specifically, I explore the degree to which BRW can form an effective bridge for blood parasites between wintering and breeding grounds, and whether parasites exhibit host and geographical shifts in transmission areas.

## Methods

### Study sites, sample collection and blood smear preparation

The wintering populations of the BRW were sampled in northern and southern India. In northern India, the BRW occurs as a passage migrant in Uttarakhand (UK) with two waves of arrival from April–May and October–November. In UK, bird sampling was conducted in the foothills of the Himalayas in Dehradun [DUN, 30.17409°N, 77.582535°E; 640 m above sea level (a.s.l.)] in April–May (*n* = 35), September–October in 2009–2010 (*n* = 13) and April–May in 2014–2016 (*n* = 25). The other two UK sites: Mandal [MAN, 30.44685°N, 79.27328°E; 1800 m a.s.l., *n* = 4] and Anusuya [ANS, 30.47888°N, 79.28503°E; 2000 m a.s.l., *n* = 7] were sampled in April–May in 2014–2016. The BRW is the most common wintering warbler in southern India where sampling was conducted from October–March in 2014–2015 in Bangalore, Karnataka [BNG, 13.320161°N, 77.381302°E; 900 m a.s.l. *n* = 72; Fig. 1).

At each location, 6–12 mist nets were set up in high bird activity locations, often along forest edges, footpaths, or off-road nature trails. Mist nets were 38-mm gauge mesh, 2.6 m tall, and 6, 9, or 12 m long. Birds were sampled birds between 0540 and 1230 and nets were checked every 5–10 min. Each bird caught was identified using Rasmussen and Anderton (2005); ringed and captured individuals were released at the site after processing. Birds were not aged as many BRW were in heavy moult (see Gaston 1976).

In total, 156 BRW were captured and 20–40 μL of bird blood was sampled from the sub-brachial wing vein (never exceeding 1% of the individual’s body weight). All samples were stored in SET Buffer (20–40 μL in 500 μL buffer 0.15 M NaCl, 0.05 M Tris, 0.001 M EDTA, pH 8.0) at room temperature and subsequently transferred to −20 °C. From 2014 to 2016, thin blood smears from 78 birds were prepared on glass slides and then air-dried, fixed in 100% methanol and stained with Giemsa.

### Molecular, morphological and phylogenetic analyses

The presence of *Plasmodium*, *Haemoproteus* and *Leucocytozoon* was assessed using parasite-specific primers designed to amplify partial fragments of the cytochrome *b* (cyt *b*) gene (see supplementary material, S1). Molecular sexing of the birds was done by PCR with the primers 0057 F and 002 R amplifying introns of the CHD1Z/W gene (Round et al. 2007). Blood smears were examined for the presence of haemosporidians and gametocytes following Godfrey et al. (1987). To explore evolutionary relationships, a model-based approach was used following phylogenetic reconstruction using the maximum likelihood analysis on the sequences isolated from the BRW in India as well as avian haemosporidian sequences (34 *Haemoproteus* and 21 *Plasmodium*) found in *Acrocephalus* warblers in Europe and central Asia as per the MalAvi database (Bensch et al. 2009). GenBank accession number are KY695225-KY695231.

### Statistical analysis

#### Parasite prevalence

Using PCR-based results, for unbiased prevalence estimates, I calculated prevalence of haemosporidians with 95% confidence intervals (95% CI) using the Sterne exact method (Reiczigel 2003) in Quantitative Parasitology, version 3.0 (Rózsa et al. 2000).

To assess variation in prevalence of common lineages (*n* ≥ 5) in birds sampled by gender and location (passage migration and wintering population), a generalised linear model (GLM) was used with the number of birds infected and number uninfected as the binomial response variable and location as the categorical explanatory variable. Data for two sampling periods (2009–2010 and 2014–2016) in UK were analysed together as there was not enough statistical power for temporal analysis. Analyses were conducted in R v. 3.0.1(R Core Team 2012).

#### Estimation of parasite lineage richness

To estimate success in sampling all available parasite lineages in wintering BRW populations, I calculated a rarefaction (species-area) curve and estimated true lineage richness with classic Chao2 estimations (Colwell 2013). I used ESTIMATES version 9.0 (Colwell 2013) to construct linked rarefaction and extrapolation curves. Parasite richness (Chao2) estimates were considered to be significantly different if 95% confidence intervals (CIs) did not overlap.

## Results

### Parasite prevalence

Avian haemosporidian infections were found in 64 out of 156 (41.02%; 95% CI 33.3–49%) individuals across all BRW populations. Of the positive samples, 37.8% (95% CI 30.41–45.82%) were *Haemoproteus* spp. and 1.9% (95% CI 0.053–5.6%) were *Plasmodium* spp. None of the samples were infected with *Leucocytozoon* spp. Prevalence of *Haemoproteus* spp. in wintering site (BNG: 60.3%; 95% CI 47.4–72.5%) and in passage migration (UK) sampled in 2009 (45%; 95% CI 29.7–61.2%), and 2014–2016 (38.5%; 95% CI 21.17–57.7%) did not show significant difference (*χ* ^2^ = 4.23, *df* = 2, *P* = 0.12). Similarly, there was no significant difference in prevalence of *Haemoproteus* spp. (GLM: *χ* ^2^ = 1.45, *P* = 0.23) by gender.

In total, 78 BRW blood smears collected, of which 75 (96%) were in agreement with PCR with medium to high intensity *Haemoproteus* (98%) and *Plasmodium* (1%) infections and three were submicroscopic. All infected smears showed the presence of both male and female gametocytes.

There were six *Haemoproteus* lineages and one *Plasmodium* lineage recovered from BRW populations (Fig. 1), of which three *Haemoproteus* lineages, ACDUM3, ACDUM4 and ACDUM5 have not been described previously (supplementary material S1, Table S1). The *Plasmodium* lineage SW2 (*n* = 3) was only found in BNG. The prevalence of lineages showed no significant difference by gender ACDUM1 (GLM: *χ* ^2^ = 0.68, *P* = 0.72), ACDUM2 (GLM: *χ* ^2^ = 0.68, *P* = 0.72) and location: ACDUM1 (GLM: *χ* ^2^ = 0.12, *P* = 0.72), ACDUM3 (GLM: *χ* ^2^ = 3.37, *P* = 0.06), MW1 (GLM: *χ* ^2^ = 0.017, *P* = 0.89), and ACDUM2 (GLM: *χ* ^2^ = 0.72, *P* = 0.39).

### Estimation of parasite lineage richness

The BRW populations in passage migration (UK) and wintering (BNG) sites showed no difference in lineage richness as evidenced by overlapping Chao2 95% CIs (Table 1). Across the entire BRW populations, the accumulation curve reached an asymptote (Sobs = 7, Chao2 = 6.14 ± 1.45; supplementary material S1, Figure S1).

**Table 1:** Lineage diversity estimates (Chao2 ± SD) across wintering populations of the Blyth’s Reed Warbler in India

Site	Year	*N*	Male/female			*M*	*N*_seq_	Sobs	Chao2	95% CI
			*N*	*H*	*P*					
Northern India: passage migrant										
DUN	2009	48	33/15	11/7	0	2	20	5	4.11 ± 1.29	3.39–10.07
DUN	2014–2016	25	21/4	6/1	0	0	7			
MAN	2016	4	3/1	1/0	0	0	1			
ANS	2016	7	6/1	2/1	0	0	0			
Total		84	63/21	20/9	0	0	8	4	3.14 ± 1.12	2.49–8.08
Southern India: wintering										
BNG	2014–2016	72	34/38	15/15	1/2	0	33	6	5.21 ± 1.18	4.58–10.61
Total		156		59	3	0	61	7	6.31 ± 1.98	5.35–16.57

*DUN*, Dehradun; *MAN*, Mandal; *ANS*, Anusuya; *BN,* Bangalore*; N*, sample size of birds screened; *H*, *Haemoproteus* infection; *P*, *Plasmodium* infection; *M*, mixed infection; *N*_*seq*_, number of infected samples that were sequenced to determine lineage identity; *Sobs*, number of lineages detected; *Chao2*, lineage diversity estimate ± standard deviation; 95% *CI*, Chao2 95% confidence interval

### Parasite lineage diversity, host and geographical shifts

**Fig. 2:**
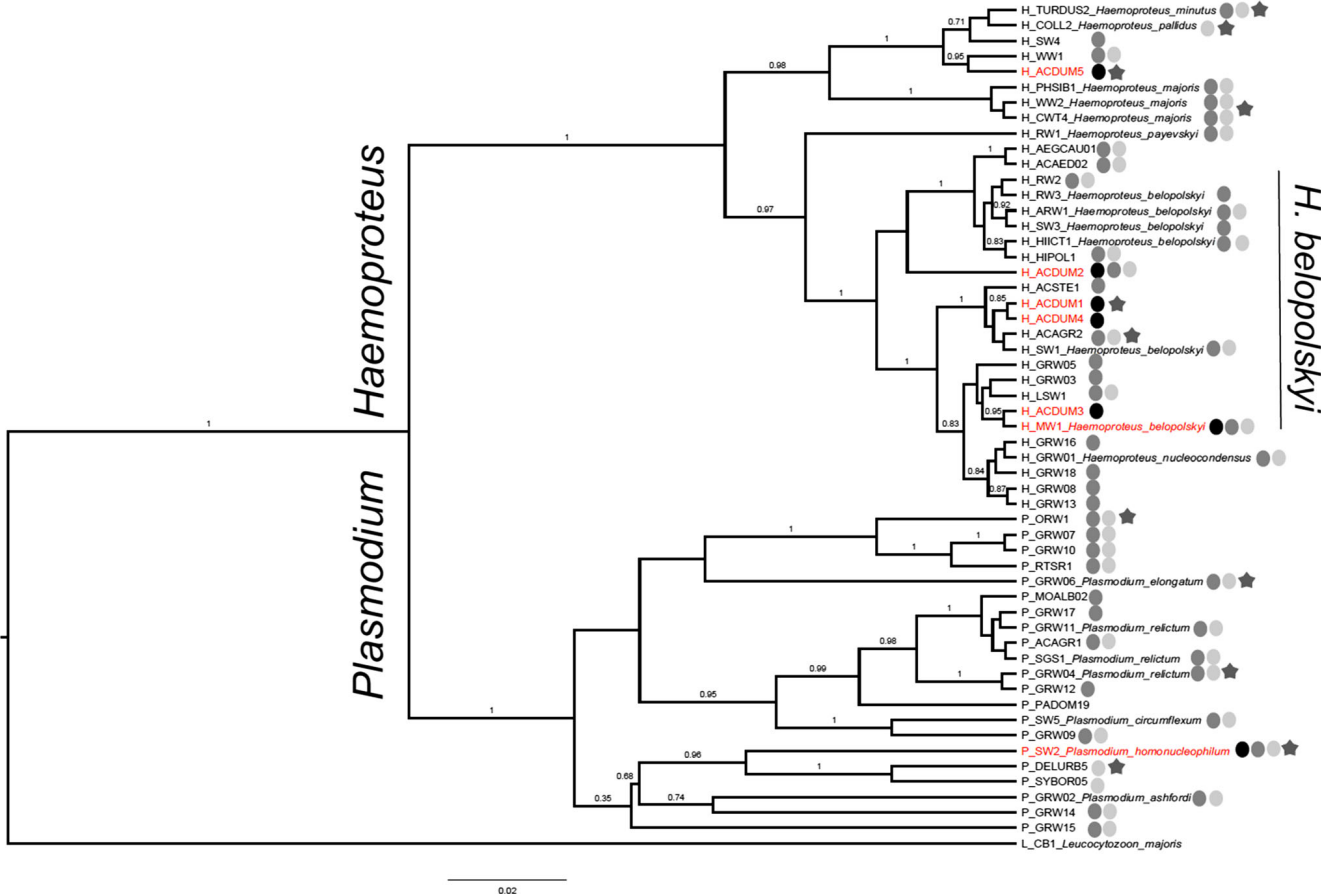
Maximum clade credibility tree of *Plasmodium* and *Haemoproteus* lineages recovered from Blyths’ Reed Warbler in India from based on cytochrome *b* gene (477 bp). Posterior clade probability support values above 0.5 are shown. Lineages found in Blyth’s Reed Warbler are in *red text* and indicated with *black oval dot*. Lineages shared with other *Acrocephalus* warblers are in *grey dot*. Lineages shared with other avian host are shown in *light* *grey dot*. Star indicates lineages shared with avian hosts in India

The parasite phylogeny included 32 *Haemoproteus* lineages and 20 *Plasmodium* lineages, of which seven lineages were found in the BRW (Fig. 2). Five *Haemoproteus* lineages in the BRW fall in the *H. belopolyski* clade. Of which three lineages ACDUM1, ACDUM2 and MW1 have been recovered in *Acrocephalus* and *Hipplolais* warblers in Europe, Russia and non-*Acrocephalus* hosts in Africa. In India, ACDUM1 was shared with a Pink-browed Rosefinch *Carpodacus rodochroa* and ACDUM5 was found in a Streaked Laughingthrush *Trochalopteron lineatum* sampled in UK. Two newly recovered *Haemoproteus* lineages showed 0.2% (corresponds to 1 bp substitution) sequence divergence between ACDUM1/ACDUM4 and MW1/ACDUM3.

## Discussion

The wintering populations of BRW harboured a *Haemoproteus* dominated parasite assemblage with absence of infectious stages in resident birds in the wintering ground. In particular, a large proportion of *Haemoproteus* lineages in *H. belopolsyki* clade appears to be a complex of recently diversified (0.2%; corresponds to 1 bp substitution) lineages within the BRW. The BRW occurs in sympatry in breeding range with *Acrocephalus agricola* (winter in south Asia) and *Acrocephalus scirpaceus* (winter in sub-Saharan Africa) which potentially facilitates the transfer of host-specific parasites in phylogenetically closely related species (see Cooper et al. 2012).

The lineages ACDUM2 and MW1 were shared only with *Acrocephalus* warblers in breeding quarters and not detected in resident birds (~3000) in India (Ishtiaq et al., unpublished) which suggest that transmission is restricted to Europe and Africa. The most prevalent lineage ACDUM1 differs by single base pair substitution with ACAGR2, a lineage common in the Paddyfield Warbler in Bulgaria and Russia (Zehtindjiev et al. 2009). However, based on a 351 bp cyt *b* identical match, both lineages have been reported in three resident avian hosts in India (Ishtiaq et al. 2007; Bensch et al. 2009). In addition, two lineages, ACDUM1and ACDUM5, were detected in resident Himalayan birds, albeit in low frequency, that point towards shift in transmission areas as India or possible spill over infections (see Hellgren et al. 2009). Whilst this may be an artefact, as the PCR technique does amplify sporozoite DNA (Valkiūnas et al. 2009), a close examination of blood smears from resident Himalayan birds confirmed the absence of gametocytes in the blood as several biotic (e.g., competent vectors) and abiotic (e.g., temperature) factors are central for parasite to complete its development in a new environment. The only detected *Plasmodium* lineage SW2 that was previously identified as *Plasmodium homonucleophilum* has been found in multiple avian hosts in Europe, Central Asia, Africa and India.

Given the limited sample size, the lineage accumulation curve reached an asymptote, and it is worth pointing out that there were generalist and widely distributed parasites lineages (e.g., GRW4, DELURB4) found in resident birds in India but not harboured in the BRW. Future study in the breeding range will help to understand the parasite selection pressure and migration strategy of the BRW wintering in India.

## Electronic supplementary material

Below is the link to the electronic supplementary material.

Supplementary material 1 (DOCX 165 kb)
