# Supplementary material for: Exploring host and geographical shifts in transmission of haemosporidians in a Palaearctic passerine wintering in India
Source: J Ornithol. 2017 Mar 9;158(3):869–74. doi: 10.1007/s10336-017-1444-9 (PMC6038909; doi:10.1007/s10336-017-1444-9)
Supplement: Supplementary file 1 — Supplementary material 1 (DOCX 165 kb) [file 10336_2017_1444_MOESM1_ESM.docx]

**Supplementary material S1:**

**Exploring host and geographical shifts in transmission of haemosporidians in a Palaearctic passerine wintering in India**

**Farah Ishtiaq**

**Centre for Ecological Sciences, Indian Institute of Science, Bangalore-560012, INDIA**

Email: Ishtiaq.farah@gmail.com

Tel: +91 80 22932507

Fax: +91 80 23601428

**Methods**

***Molecular analyses***

DNA extractions were conducted using Phenol Chloroform extraction method (Sambrook *et al*. 1989). DNA samples were screened for the presence of parasite infection using a restriction enzyme-based assay designed to amplify a 160 bp fragment of mitochondrial ribosomal RNA gene (rRNA) of avian haemosporidians (Beadell and Fleischer 2005). For samples that screened positive for the 16S rRNA gene fragment of the parasites, the cytochrome *b* (cyt b) gene fragments for *Plasmodium* and *Haemoproteus* were amplified ranging from 533bp, 477bp following Beadell *et al*. (2004) and Hellgren *et al*. (2004) respectively. All samples were screened for the presence of *Leucocytozoon* using HaemFL/HaemR2L (478 bp) following Hellgren *et al*. (2004). Each plate accompanied parasite positive and negative controls to examine the potential contamination. Parasite negative samples were screened for bird cyt *b* gene following Dumbacher *et al*. (2003). The resulting PCR products were then sequenced in both directions. Sequences were assembled, aligned and edited using SEQUENCHER version 5.2. I then identified sequences to genus to their closest sequence matches in GenBank or MalAvi databases (Bensch *et al*. 2009).

***Phylogenetic analysis***

To explore evolutionary relationships, a model-based approach was used following phylogenetic reconstruction using the maximum likelihood analysis on the sequences isolated from the BRW in India as well as avian haemosporidian sequences (34 *Haemoproteus* and 21 *Plasmodium*) found in *Acrocephalus* warblers in Europe and central Asia as per MalAvi database (Bensch *et al*. 2009). The maximum likelihood tree was constructed using Bayesian phylogenetics as implemented in BEAST version 1.4.3 (Drummond *et al*. 2006) using the most appropriate substitution model (GTR+G) according to the Akaike Information Criterion implemented MEGA version 5.2 (Tamura *et al*. 2011). I present a Maximum Clade Credibility (MCC) tree using a relaxed molecular clock approach (Drummond *et al*. 2006). Rates of substitution were drawn from a lognormal distribution and Yule prior was used for branching rates. I conducted two runs of 20 million generations, each with sampling conducted every 1,000 generations. *Tracer* (Rambaut and Drummond, 2003) was used to assess convergence, whether two chains were mixing and whether the estimated sample size (ESS) for each parameter was of sufficient size (ESS>200) to obtain robust parameter estimates. Four million generations were discarded as burn-in from each run, leaving a posterior distribution of 32,000 trees.

References

Beadell JS, Fleischer RC (2005) A restriction enzyme-based assay to distinguish between avian hemosporidians. J Parasitol 91: 683-685

Beadell JS, Gering E, Austin J, Dumbacher JP, Peirce MA, Pratt TK, Atkinson CT, Fleischer RC (2004) Prevalence and differential host-specificity of two avian blood parasite genera in the Australo-Papuan region. Mol Ecol 13: 3829–3844

Drummond AJ, Rambaut A (2007) BEAST: Bayesian evolutionary analysis by sampling trees. BMC Evol. Biol. 7.1:1

Dumbacher JP, Pratt TK, Fleischer RC (2003) Phylogeny of the owlet-nightjars (Aves: Aegothelidae) based on mitochondrial DNA sequence. Mol. Phyl. Evol. 29: 540–549.

Hellgren O, Waldenstrom J, Bensch S (2004) A new PCR assay for simultaneous studies of *Leucocytozoon*, *Plasmodium*, and *Haemoproteus* from avian blood. J Parasitol 90: 797−802

Rambaut A, Drummond AJ (2003) Tracer v1.3, available from <http://evolve.zoo.ox.ac.uk/>.

Sambrook J, Fritsch EF, Maniatis T (1989) Molecular Cloning- A Laboratory Manual. Cold Spring Harbor, New York

Tamura K, Peterson D, Peterson N, Stecher G, Nel M, Kumar S (2011) MEGA5: Molecular Evolution Genetics Analysis using Maximum Likelihood, Evolutionary Distance, and Maximum Parsimony Methods. Mol Biol Evol 28: 2731-2739

**FIGURE S1**. Sample-based rarefaction and extrapolation curves of parasite lineage diversity for all lineages combined around a sample reference point with 95% unconditional confidence intervals.


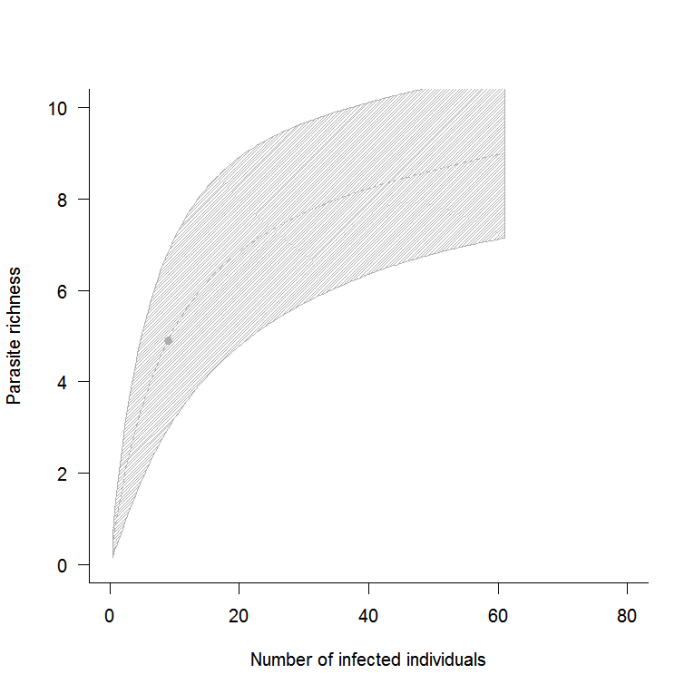


Table S1 Host ranges of parasite lineages and additional host–parasite occurrences in the Blyth’s Reed Warbler

*P, *Plasmodium*; H, *Haemoproteus; newly found lineages in Blyth’s reed warbler are marked in Bold. ª also matches with ACAGR2*

**References**

Bensch S, Hellgren O, Pérez‐tris J (2009) MalAvi: a public database of malaria parasites and related haemosporidians in avian hosts based on mitochondrial cytochrome *b* lineages. Mol Ecol Resour 9: 1353-1358.

Hellgren O, Waldenström J, Pérez-Tris J, Szollosi E, Hasselquist D, Križanauskienė A, Ottosson U, Bensch S (2007) Detecting shifts of transmission areas in avian blood parasites−a phylogenetic approach. Mol Ecol 16:1281–1290.

Ishtiaq F, Gering E, Rappole JH, Rahmani AR, Jhala YV et al. (2007) Prevalence and diversity of avian hematozoan parasites in Asia: a regional survey. J Wildl Dis 43: 382-398.

Dimitrov D, Zahtindjiev P, Bensch S (2010) Genetic diversity of avian blood parasites in SE Europe: cytochrome b lineages of the genera *Plasmodium* and *Haemoproteus* (Haemosporida) from Bulgaria. Acta Parasitologica 55: 201–209.

Reullier J, Pérez-Tris J, Bensch S, Secondi J (2006) Diversity, distribution and exchange of blood parasites meeting at an avian moving contact zone. Molecular Ecology 15: 753–763.
